# Deficiency of S100A9 Alleviates Sepsis-Induced Acute Liver Injury through Regulating AKT-AMPK-Dependent Mitochondrial Energy Metabolism

**DOI:** 10.3390/ijms24032112

**Published:** 2023-01-20

**Authors:** Yanting Zhang, Feng Wu, Fei Teng, Shubin Guo, Huihua Li

**Affiliations:** Beijing Key Laboratory of Cardiopulmonary Cerebral Resuscitation, Department of Emergency Medicine, Beijing Chaoyang Hospital, Capital Medical University, Beijing 100020, China; yanting953@mail.ccmu.edu.cn (Y.Z.); fengwu@mail.ccmu.edu.cn (F.W.); tengfei.vip@139.com (F.T.)

**Keywords:** S100A9, sepsis, acute liver injury, AMPK, mitochondrial energy metabolism

## Abstract

Acute liver injury (ALI) is recognized as a serious complication of sepsis in patients in intensive care units (ICUs). S100A8/A9 is known to promote inflammation and immune responses. However, the role of S100A8/A9 in the regulation of sepsis-induced ALI remains known. Our results indicated that S100A8/A9 expression was significantly upregulated in the livers of septic mice 24 h after cecal ligation and a puncture (CLP) operation. Moreover, S100A9-KO in mice markedly attenuated CLP-induced liver dysfunction and injury, promoting the AMPK/ACC/GLUT4-mediated increases in fatty acid and glucose uptake as well as the improvement in mitochondrial function and ATP production. In contrast, treatment with the AMPK inhibitor Compound C reversed the inhibitory effects of S100A9 KO on CLP-induced liver dysfunction and injury in vivo. Finally, the administration of the S100A9 inhibitor Paquinimod (Paq) to WT mice protected against CLP-induced mortality, liver injury and mitochondrial dysfunction. In summary, our findings demonstrate for the first time that S100A9 plays an important pro-inflammatory role in sepsis-mediated ALI by regulating AKT-AMPK-dependent mitochondrial energy metabolism and highlights that targeting S100A9 may be a promising new approach for the prevention and treatment of sepsis-related liver injury.

## 1. Introduction

Sepsis is a common complication after infection, shock and severe trauma, and it is one of the major causes of mortality in patients in intensive care units (ICUs) [1,2]. Upon pathogen infection, the host innate immune system is excessively activated; innate immune activation is followed by and causes the occurrence of a cytokine storm, which is characterized by high levels of pro-inflammatory cytokines, including TNF-α, IL-1β, IL-6, and IFN-γ, and results in cell death and multiple organ dysfunction [1,2]. Importantly, the liver is the main target of the inflammatory response, and it is one of the most severely affected organs in sepsis. Acute liver injury (ALI) is recognized as an important cause of mortality in patients with sepsis [1,2]. The main manifestations of sepsis-induced liver dysfunction consist of hypoxic hepatitis, cholestasis, and impairment of metabolism-related protein synthesis and coagulopathy [3]. Unfortunately, no effective therapeutic approaches are currently available to cure this disease, and the pathophysiology of the disease is still not fully understood.

Increasing amounts of evidence suggest that the elevation of pro-inflammatory cytokines is closely associated with sepsis-associated liver dysfunction. S100 calcium binding protein A8 (S100A8) and A9 (S100A9) (also known as MRP-8/14, respectively) belong to the S100 family of calcium binding proteins and can form heterodimers, which function as danger-associated molecular patterns (DAMPs, also called alarmins) and display some functional features of pro-inflammatory cytokines [4]. Multiple studies have shown that S100A8/A9 are upregulated in the circulation of animals and patients with sepsis, and that their levels are associated with disease severity [5]. Moreover, S100A8/A9 has been demonstrated to inhibit cell growth, cause cytotoxicity and induce apoptosis through the activation of the danger signal receptors such as Toll-like receptor 4 (TLR4) or the receptors for advanced glycation end-products (RAGE), thereby eliciting a variety of inflammatory responses and causing endotoxin-induced multiple organ failure [4,6]. Several studies have revealed that S100A8/A9 displays a critical role in the pathogenesis of different diseases [4]. Recent studies indicate that S100A8/A9 could be a valuable biomarker for the diagnosis and prognosis of many inflammatory and autoinflammatory diseases, such as Crohn’s disease, rheumatoid arthritis, bowel disease, and ischemic stroke, and that its expression is positively associated with adverse clinical outcomes in these diseases [7,8,9,10]. However, the functional role of S100A8/A9 in sepsis-induced acute liver injury (ALI) and whether it exerts a therapeutic effect on this disease remain elusive.

Since deletion of the S100A8 gene in mice is lethal, and S100A9-deficient mice exhibit a healthy phenotype but have no S100A8 protein express [11]. Moreover, S100A9 is required for the stability of S100A8 protein and S100A8/A9 function [12]. Therefore, here we mainly investigate the role of S100A9 in cecal ligation and puncture (CLP)-induced ALI in S100A9-knockout (KO) mice or wild-type (WT) mice treated with the S100A9 inhibitor Paquinimod (Paq). Our results indicate that the S100A8 and S100A9 protein levels are significantly upregulated in the liver of mice after CLP operation. The ablation or inhibition of S100A9 markedly attenuated CLP-induced hepatic injury and dysfunction, and these effects were accompanied by the inhibition of AKT and the activation of an AMPK-dependent increase in mitochondrial energy metabolism. However, inhibition of AMPK abolished these beneficial effects in CLP-treated S100A9-KO mice. Thus, our data provide novel insights into the mechanism underlying sepsis-induced liver injury by linking S100A9 to AKT-AMPK-mitochondrial energy metabolism. This study also identifies S100A9 as a potential therapeutic target for treating sepsis-associated liver injury.

## 2. Results

### 2.1. S100A8 and S100A9 Expression Is Upregulated in the Livers of CLP-Treated Mice

**Figure 1:**
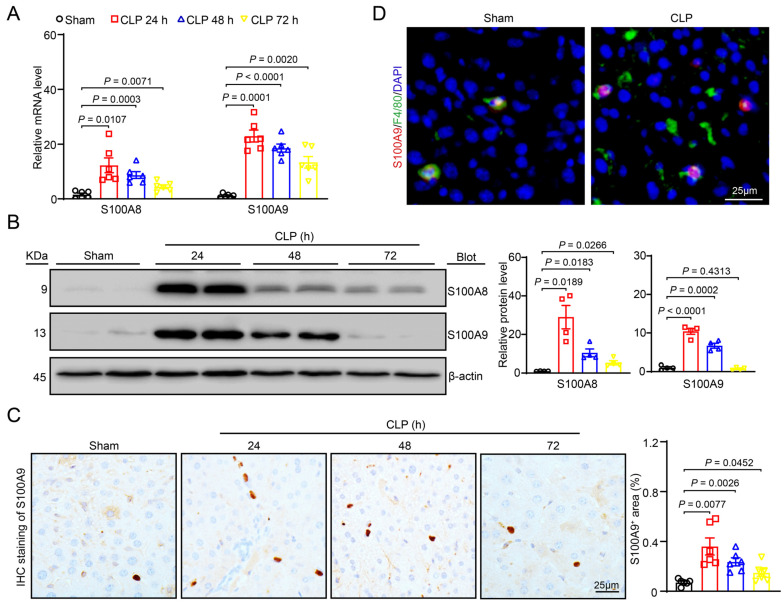
Upregulation of S100A8/A9 in the liver of CLP-treated mice. Polymicrobial sepsis in wild-type (WT) mice was induced by cecal ligation and puncture (CLP) for 24–72 h. Sham group: 24 h. (**A**) qPCR assay of S100A8 and S100A9 mRNA levels in the liver at each time point (*n* = 6). (**B**) Immunoblotting analysis of S100A8 and S100A9 protein levels in the liver at each time point (left), and quantification of the relative protein level (right, *n* = 4). (**C**) Immunohistochemical staining of liver sections with anti-S100A9 antibody (left, brown area), and quantification of the S100A9^+^ area (right, *n* = 6). (**D**) Immunofluorescent staining of liver sections with anti-F4/80 (green) or anti-S100A9 (red) antibody to examine localization of S100A9 in F4/80^+^ macrophages. Data are shown as mean ± SEM, and *n* represents the number of mice in each group.

To determine the expression of S100A8/A9 in the livers of CLP-treated mice, we first established a sepsis-associated ALI model via CLP. qPCR assays indicated that the S100A8 and S100A9 mRNA levels were significantly enhanced in the livers at 24, 48 and 72 h after CLP compared with sham group (24 h) (Figure 1A). Immunoblotting demonstrated that the protein levels of S100A8 and S100A9 peaked at 24 h and then decreased until 72 h (Figure 1B). Immunohistochemical staining with anti-S100A9 antibodies confirmed the increased S100A9 expression in the livers of CLP-treated mice (Figure 1C) and that S100A9 colocalized with F4/80-positive macrophages in the liver tissues (Figure 1D).

### 2.2. Knockout of S100A9 Attenuates Susceptibility to CLP-Induced Acute Liver Injury

**Figure 2:**
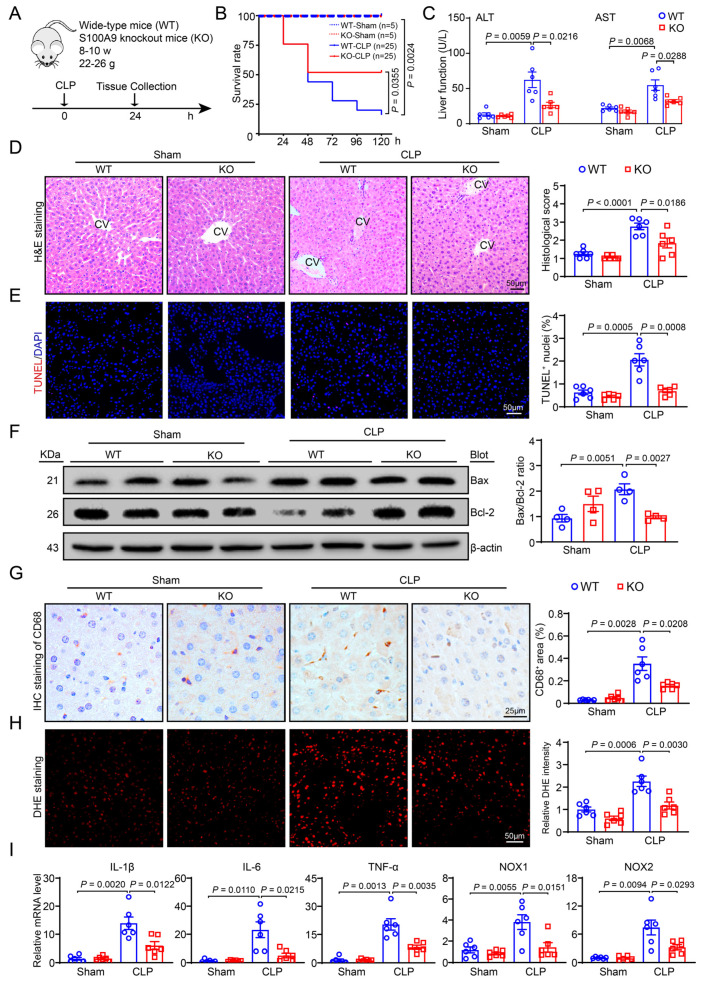
Knockout of S100A9 in mice suppresses CLP-induced liver dysfunction, hepatocyte damage, apoptosis, inflammation and superoxide production. (**A**) Schematic diagram of WT or S100A9-knockout (KO) mice subjected to CLP for 24 h. (**B**) Survival rate of WT and S100A9 KO mice after sham or CLP operation (sham group, *n* = 5 per group; CLP group, *n* = 25 per group). (**C**) The levels of serum ALT and AST in each group (*n* = 6). (**D**) H&E staining of liver sections (left, CV: central veins), and quantification of the histological score (right, *n* = 6). (**E**) TUNEL (red) and DAPI (blue) staining of liver sections (left), and quantification of the TUNEL^+^ nuclei (right, *n* = 6). (**F**) Immunoblotting assay of Bax and Bcl-2 protein levels (left), and the ratio of Bax to Bcl-2 (right, *n* = 4). (**G**) Immunohistochemical staining of liver sections using anti-CD68 antibody (left, brown area), and quantification of the CD68^+^ area (right, *n* = 6). (**H**) Dihydroethidium (DHE) staining of liver sections to measure superoxide production (left) and quantification of fluorescence intensity (right, *n* = 6). (**I**) qPCR assay of IL-1β, IL-6, TNF-α, NOX1 and NOX2 mRNA levels (*n* = 6). Data are shown as mean ± SEM, and *n* represents the number of mice in each group.

To examine the role of S100A9 in ALI in a mouse model of sepsis, we generated S100A9-knockout (KO) mice and subjected them to CLP (Figure 2A). Five days after CLP, the survival rate was significantly higher in S100A9-KO mice (52%, 4/25) than in WT mice (16%, 13/25) (Figure 2B). Moreover, 24 h after CLP, marked liver dysfunction was observed, as indicated by increased serum AST and ALT levels in WT mice compared with sham control mice. However, this effect was greatly ameliorated in S100A9-KO mice (Figure 2C). H&E analysis revealed that CLP-treated S100A9-KO mice had fewer pathological changes in the liver, including inflammation, necrosis, and thrombus formation, than CLP-treated WT mice 24 h after CLP (Figure 2D). Furthermore, TUNEL staining revealed that the CLP-induced increase in hepatic cell apoptosis was substantially/markedly lower in S100A9-KO mice than in WT mice (Figure 2E). Accordingly, the CLP-induced increase in the Bax/Bcl-2 ratio in the hepatic tissues of WT mice was markedly reversed in S100A9-KO mice (Figure 2F).

### 2.3. Deficiency of S100A9 Suppresses CLP-Induced Hepatic Inflammation and Oxidative Stress

Next, we investigated whether S100A9 KO affects CLP-induced hepatic inflammation and ROS production, both of these effects being associated with sepsis-induced ALI. Twenty-four hours after CLP, S100A9-KO mice showed marked decreases in CD68-positive macrophage infiltration and ROS production (DHE staining) in their livers compared with the WT mice (Figure 2G,H). Accordingly, qPCR assays indicated that the mRNA levels of IL-1β, IL-6 and TNF-α and NADPH oxidase isoforms (NOX1 and NOX2) in the livers were significantly lower in S100A9-KO mice than in WT mice after CLP (Figure 2I). No differences in these parameters were observed between the WT and S100A9-KO groups after sham operation (Figure 2B–I).

### 2.4. Knockout of S100A9 Activates AMPK in CLP-Induced Hepatic Injury

**Figure 3:**
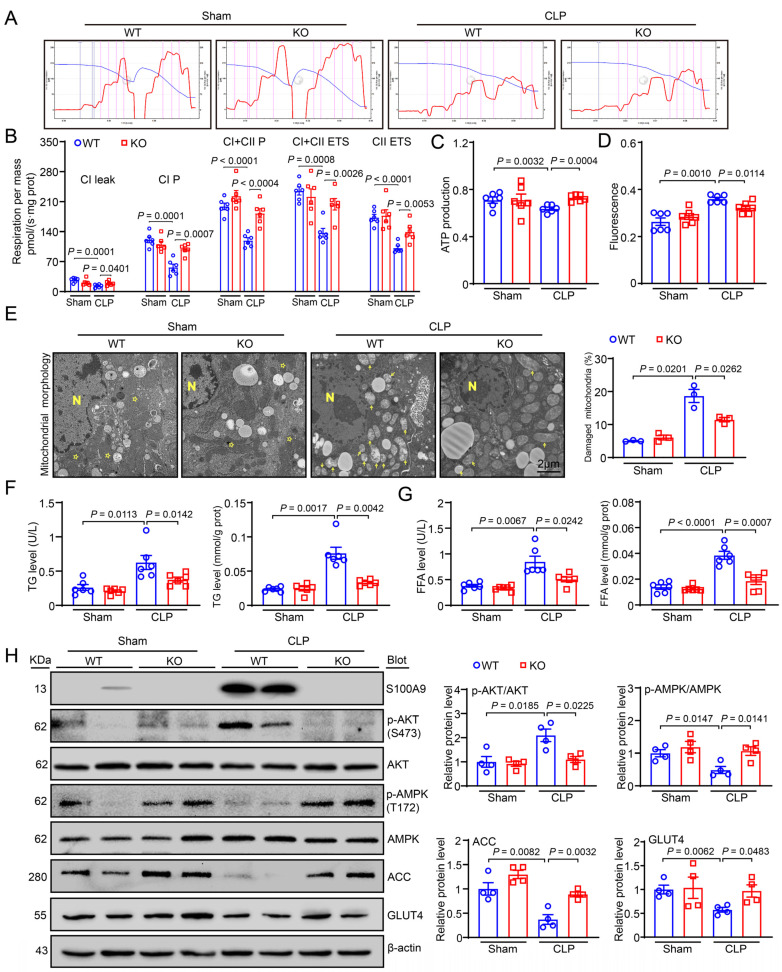
Knockout of S100A9 ameliorates liver mitochondrial dysfunction through increasing AMPK-medaited glucose and lipid metabolism. (**A**) Representative images of O_2_ concentration change (blue line) and O_2_ flux per mass (red line) in groups. (**B**) Summarized data for the oxygen consumption capacity measured by high-resolution respirometry in CI leak, CI P (complex I OXPHOS), CI + CII P, CI + CII ETS (electron transfer system capacity), and CII ETS (*n* = 6). (**C**) ATP production and (**D**) fluorescence (represent mitochondrial membrane potential) (*n* = 6). (**E**) TEM of liver tissue (left) and the percentage of damaged mitochondria (right, *n* = 3). Asterisk: normal mitochondria; Arrowheads: damaged mitochondria; *N*: nucleus; magnification is 2000×. (**F**) Serum and (**G**) tissue levels of TG and FFA (*n* = 6). (**H**) Immunoblotting analysis of S100A9, p-AKT, AKT, p-AMPK, AMPK, ACC, GLUT4 (left) and quantification of the relative protein levels (right, *n* = 4). Data are shown as mean ± SEM, and *n* represents the number of mice in each group.

Mitochondrial dysfunction frequently triggers the apoptosis of live cells [13]. Therefore, we examined the effect of S100A9 KO on mitochondrial respiration in liver tissues using the Oroboros Oxygraph-2k respirometer (Innsbruck, Austria). A high-resolution respiratory assay showed that both the mitochondrial oxidative phosphorylation (OXPHOS) capacity and electron transfer system (ETS) activity of complexes I and II, as indicated by CI leak, CI- and CI plus CII-OXPHOS, CI plus CII- and CII-ETS, were significantly inhibited in the livers of CLP-treated WT mice compared with the livers of sham-treated mice. However, this decrease was greatly ameliorated in CLP-treated S100A9-KO mice (Figure 3B). Additionally, ATP production and mitochondrial membrane potential (MMP) were lower in the livers of CLP-treated WT mice, and these effects were reversed in the livers of CLP-treated KO mice (Figure 3C,D). In addition, TEM analysis showed a several damaged mitochondria in WT mice after CLP, as characterized by swollen organelles with distorted cristae and translucent matrix, while the changes in morphology were improved in CLP-treated KO mice (Figure 3E). All of these results indicate that S100A9 mainly impairs the function of mitochondrial respiratory complex I/II in the livers of septic mice.

Since AMP-activated protein kinase (AMPK) is a critical regulator of glucose and fatty acid (FA) metabolism and exerts a key role in protection against sepsis-induced hepatic injury [14], we then examined whether S100A9 KO triggers the activation of AMPK and mitochondrial energy metabolism. Immunoblotting indicated that CLP led to a marked increase in phosphorylated (p)-AKT (S473) and a decrease in p-AMPK (T172), ACC (a key modulator of FA oxidation) and GLUT4 (a critical regulator of glucose uptake) in the livers of WT mice, and these changes were effectively reversed in the livers of S100A9-KO mice (Figure 3H). Accordingly, CLP-induced upregulation of triglyceride (TG) and free fatty acid (FFA) levels in the serum and liver tissues of WT mice was also markedly reduced in S100A9-KO mice (Figure 3F,G), suggesting that S100A9 increases the metabolism of TG and FFA by activating AKT and suppressing AMPK-ACC-GLUT4 signaling.

### 2.5. An Inhibitor of AMPK Exacerbates CLP-Induced Liver Injury in S100A9-KO Mice

**Figure 4:**
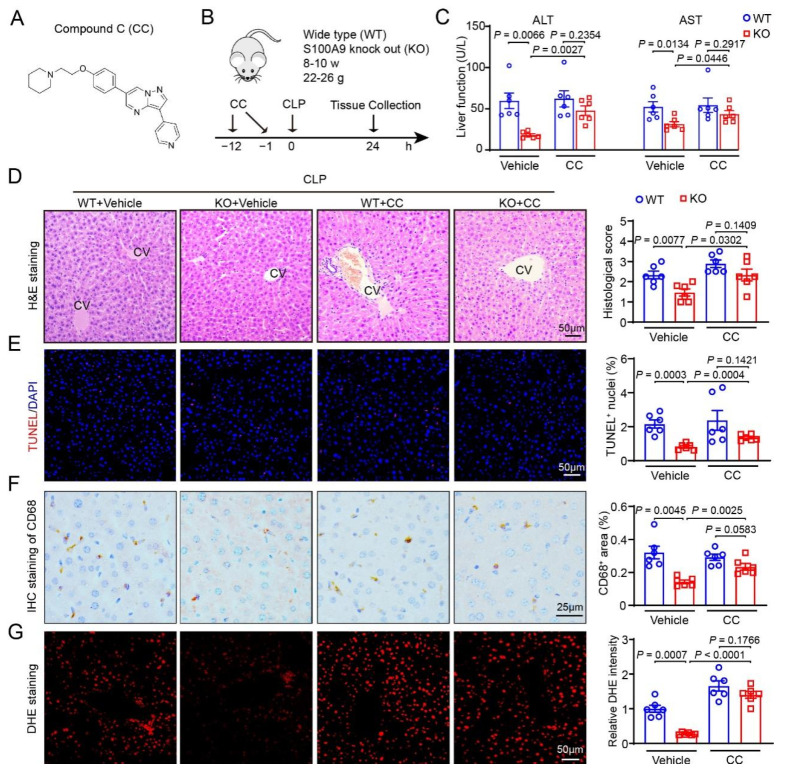
Inhibition of AMPK reverses S100A9-KO-mediated protection of CLP-induced liver dysfunction. (**A**) Diagram of AMPK inhibitor Compound C (CC). (**B**) Schematic diagram of WT or S100A9-knockout (KO) mice treated with CC and CLP operation for 24 h. (**C**) The levels of serum ALT and AST in each group (*n* = 6). (**D**) H&E staining of liver sections (left), and quantification of the histological score (right, *n* = 6). (**E**) TUNEL (red) and DAPI (blue) staining of liver sections (left), and quantification of TUNEL^+^ nuclei (right, *n* = 6). (**F**) Immunohistochemical staining of liver sections with anti-CD68 antibody (left), and quantification of the CD68^+^ area (right, *n* = 6). (**G**) DHE staining of liver sections to measure superoxide production (left), and quantification of fluorescence intensity (right, *n* = 6). Data are shown as mean ± SEM, and *n* represents the number of mice in each group.

To understand how S100A9 deficiency ameliorates ALI, we next explored the influence of the AMPK inhibitor Compound C (CC) on CLP-induced acute hepatic injury in WT and S100A9-KO mice. Consistent with the results, S100A9-KO mice exhibited significantly alleviated liver dysfunction (AST and ALT levels), necrosis, apoptosis (TUNEL^+^ nuclei numbers), inflammatory responses (infiltration of CD68^+^ macrophages), and ROS production (DHE staining) compared with WT mice after CLP (Figure 4C–G, Lane 2 versus 1). However, these inhibitory effects were greatly reversed in S100A9-KO mice treated with CC (Figure 4C–G, Lane 4 versus 2). Besides, there was no difference between WT mice and S100A9-KO mice after the administration of Compound C (Figure 4C–G, Lane 3 versus 4).

**Figure 5:**
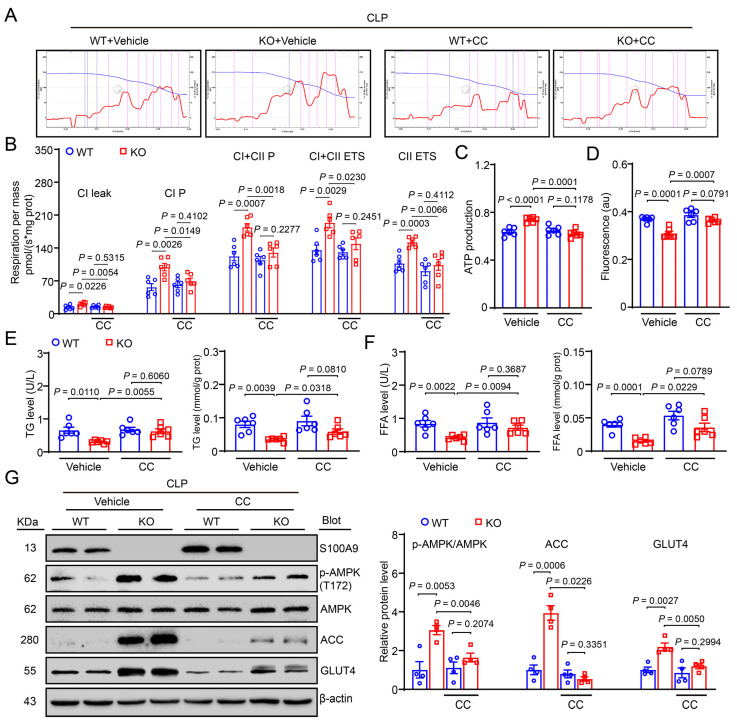
Blocking AMPK abrogates the S100A9-KO-mediated improvement of CLP-induced liver mitochondria dysfunction and reduction of glucose and lipid metabolism. (**A**) Representative images of O_2_ concentration change (blue line) and O_2_ flux per mass (red line) in groups. (**B**) Summarized data for the oxygen consumption capacity measured by high-resolution respirometry in CI leak, CI P, CI + CII P, CI + CII ETS, CII ETS (*n* = 6). (**C**) ATP production and (**D**) fluorescence (represent mitochondrial membrane potential) (*n* = 6). (**E**) Serum and (**F**) tissue levels of TG and FFA (*n* = 6). (**G**) Immunoblotting analysis of S100A9, p-AMPK, AMPK, ACC, GLUT4 (left) and quantification of the relative protein levels (right, *n* = 4). Data are shown as mean ± SEM, and *n* represents the number of mice in each group.

Next, we examined the effect of AMPK inhibition on mitochondrial respiration in liver tissues of S100A9-KO mice with the Oroboros Oxygraph-2k respirometer. The results revealed that S100A9-KO exhibited increased mitochondrial energy metabolism (both the mitochondrial OXPHOS capacity and ETS activity of complexes I and II), ATP production and MMP compared with WT mice. However, this effect was greatly reduced in CC-treated S100A9-KO mice, and there was no significant difference between CC-treated WT mice and S100A9-KO mice (Figure 5B–D). Together, these data indicate that S100A9 KO mainly improves the function of mitochondrial respiratory complex I/II in the livers of septic mice, but the inhibition of AMPK could partially exacerbate this effect. In addition, immunoblotting indicated that S100A9 KO led to a marked increase in p-AMPK (T172), ACC and GLUT4, and these changes were reduced in CC-treated S100A9-KO mice (Figure 5G). Triglyceride (TG) and free fatty acid (FFA) levels in blood and tissues showed the same results (Figure 5E,F). Collectively, these results indicate that S100A9 KO attenuates CLP-induced liver injury, possibly through the activation of an AMPK-dependent mechanism in vivo.

### 2.6. Administration of Paquinimod Reduces CLP-Induced Liver Dysfunction and Injury

**Figure 6:**
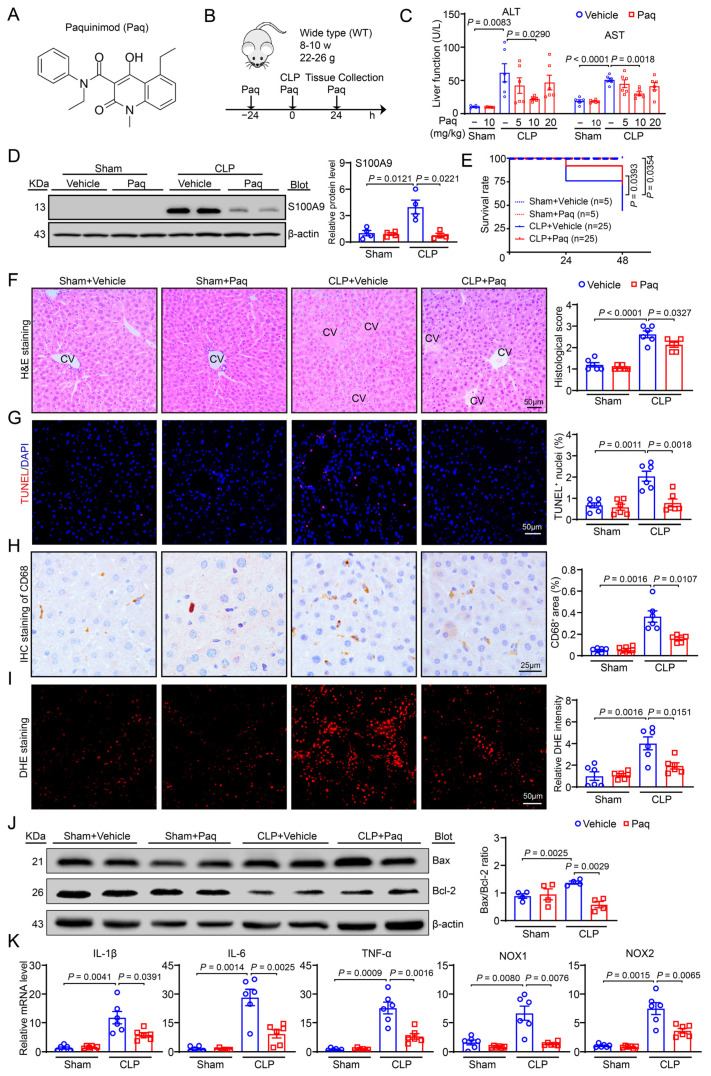
Administration of S100A9 inhibitor Paquinimod suppresses CLP-induced liver dysfunction, damage, apoptosis, inflammation and superoxide production. (**A**) Diagram of S100A9 inhibitor Paquinimod (Paq). (**B**) Schematic diagram of wild-type (WT) treated with S100A9 inhibitor (Paq) and CLP operation for 24 h. (**C**) The levels of serum ALT and AST in each group treated with Paq at dosages of 5, 10 and 20 mg/kg (*n* = 6). (**D**) The S100A9 protein level of each group treated with vehicle or Paq at dose of 10 mg/kg. (**E**) Survival rate in vehicle- or Paq-administered mice after 24 h of sham or CLP operation (sham group, *n* = 5 per group; CLP group, *n* = 25 per group). (**F**) H&E staining of liver sections (left, CV: central veins), and quantification of the histological score (right, *n* = 6). (**G**) TUNEL (red) and DAPI (blue) staining of liver sections (left), and quantification of TUNEL^+^ nuclei (right, *n* = 6). (**H**) Immunohistochemical staining of liver sections with anti-CD68 antibody (left, brown area), and quantification of the CD68^+^ area (right, *n* = 6). (**I**) DHE staining of liver sections to measure superoxide production (left) and quantify fluorescence intensity (right, *n* = 6). (**J**) Immunoblotting analysis of Bax and Bcl-2 protein levels (left), and quantification of Bax to Bcl-2 ratio (right, *n* = 4). (**K**) qPCR analysis of IL-1β, IL-6, TNF-α, NOX1 and NOX2 mRNA levels (*n* = 6). Data are shown as mean ± SEM, and *n* represents the number of mice in each group.

To demonstrate the potential clinical translational significance of our findings, we systemically administered the S100A9 inhibitor Paquinimod (Paq) to WT mice at doses of 5–20 mg/kg, subjected them to CLP, and examined them 24 h later (Figure 6A,B). Our results revealed that the CLP-induced increase in the serum AST and ALT levels was most effectively inhibited by 10 mg/kg Paq, which had no significant effects on the AST and ALT levels after sham surgery (Figure 6C). Thereafter, 10 mg/kg Paq was used to treat mice for 24 h prior to the CLP procedure. Indeed, Paq administration for 24 h almost completely abolished the CLP-induced upregulation of S100A9 protein level in the livers of mice (Figure 6D) and significantly increased the survival rates of mice (44%, 11/25) compared with vehicle specimen (72%, 18/25) 48 h after the CLP operation (Figure 6E).

Moreover, we assessed the effect of a S100A9 inhibitor (Paq) on CLP-induced liver dysfunction and injury. Our results revealed that Paq (10 mg/kg) administration significantly blocked the severity of CLP-induced hepatic injury, as indicated by necrosis, the percentage of TUNEL^+^ hepatic nuclei, infiltration of proinflammatory cells, especially CD68-positive macrophages, and ROS production, compared with vehicle administration (Figure 6F–I). Consistent with these pathological changes, the livers of vehicle-treated mice showed a marked upregulation in the Bax/Bcl-2 ratio and the mRNA levels of IL-1β, IL-6, TNF-α, NOX1, and NOX2, whereas this increase was markedly attenuated in the livers of Paq-treated mice (Figure 6J,K). There were similar parameters in the liver function or histological changes between the vehicle- and Paq-treated groups after the sham operation (Figure 6C,F–K). Overall, these findings indicate that targeting S100A9 may be a new therapeutic option for treating septic liver injury in vivo.

### 2.7. Administration of Paquinimod Improves CLP-Induced Liver Mitochondrial Dysfunction

**Figure 7:**
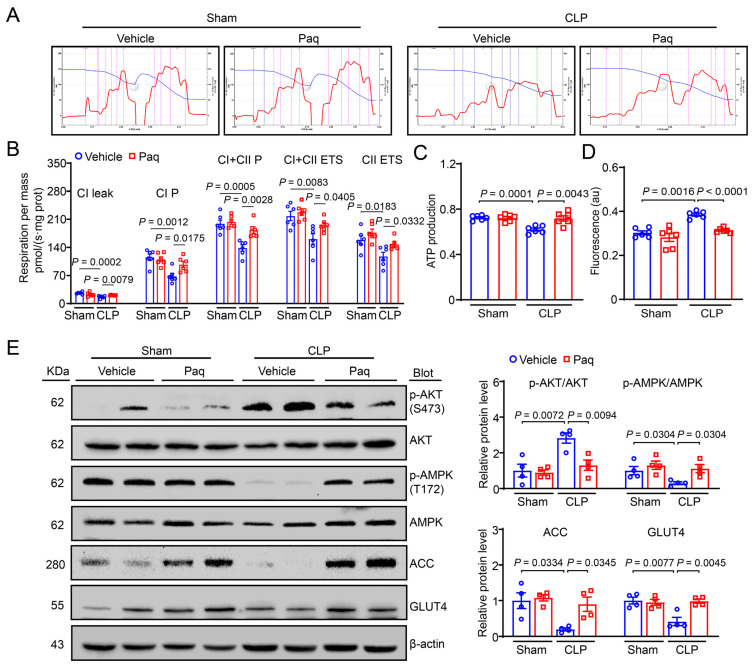
Administration of S100A9 inhibitor ameliorates liver mitochondrial dysfunction through increasing AMPK-mediated glucose and lipid metabolism. (**A**) Representative images of O_2_ concentration change (blue line) and O_2_ flux per mass (red line) in groups. (**B**) Summarized data for the oxygen consumption capacity measured by high-resolution respirometry in CI leak, CI P, CI + CII P, CI + CII ETS, CII ETS (*n* = 6). (**C**) ATP production and (**D**) fluorescence (represent mitochondrial membrane potential) (*n* = 6). (**E**) Immunoblotting analysis of p-AKT, AKT, p-AMPK, AMPK, ACC, GLUT4 (left) and quantification of the relative protein levels (right, *n* = 4). Data are shown as mean ± SEM, and *n* represents the number of mice in each group.

There were also changes in mitochondrial respiration function in liver tissues after Paq administration. The results showed that mitochondrial energy metabolism, ATP production and MMP were markedly higher in Paq-administered mice than in vehicle mice after CLP (Figure 7B,D). Then, we measured the protein levels of p-AKT, p-AMPK, ACC and GLUT4 by immunoblotting analysis, and found that the levels of these proteins were similar with those in S100A9-KO mice (Figure 7E). Collectively, these results indicate that the S100A9 inhibitor Paq attenuates the CLP-induced liver mitochondrial dysfunction, possibly through the inhibition of AKT and activation of an AMPK-dependent mechanism in vivo.

## 3. Discussion

This study reveals a previously unknown role of S100A9 in accelerating sepsis-induced hepatic dysfunction and injury. We provided compelling evidence that S100A8 and S100A9 expression was significantly elevated in the livers of CLP-operated mice. The knockout of S100A9 markedly ameliorated CLP-induced liver dysfunction, hepatocyte apoptosis, inflammation, and oxidative stress partially by blocking AKT and activating AMPK-dependent mitochondrial metabolism. Furthermore, we demonstrated that administration of the S100A9 inhibitor Paq to mice also prevented sepsis-induced liver dysfunction upon CLP operation. Therefore, this study shows that S100A9 is a critical contributor to septic liver dysfunction and highlights S100A9 as a potential therapeutic target for treating sepsis and liver dysfunction.

The S100A8 and S100A9 genes are located on chromosome 1q21, and the deletion or mutation of these chromosomes is associated with the occurrence of cancer. The human chromosomes S100A8 and S100A9 are composed of 93 and 113 amino acids with molecular masses of 10.8 and 13.2 kDa, respectively [12]. S100A8/A9 often exists in the form of a bioactive heterodimer, and its expression is highly upregulated in various tissues and cell types through different mechanisms [4]. For example, S100A8/A9 is constitutively expressed in monocytes/macrophages and neutrophils, partially through the histone methyltransferases SMYD3 and SET7/9 [4,14]. Moreover, S100A8/A9 is the most highly upregulated gene in the early stage of cardiac reperfusion [15]. Treatment of mice with ischemia or angiotensin II also markedly increases the recruitment of neutrophils to the heart, thereby producing large amounts of S100A8/A9 [16,17]. Furthermore, S100A8 and S100A9 levels are highly expressed in LPS-treated cardiomyocytes and mouse hearts [18]. Interestingly, plasma S100A9 levels are highly upregulated in CLP-treated mice [19]. More recently, a study reported that the S100A8 level is robustly upregulated in both SARS-CoV-2 animal models and COVID-19 patients [20]. In this study, we further characterized the expression pattern of S100A8/A9 in the livers of CLP-treated mice. Our data showed that both S100A8 and S100A9 expression were highly upregulated in the liver tissues of CLP-treated mice (Figure 1), suggesting that elevated S100A8/A9 may be involved in sepsis-induced acute liver injury. It has been reported that S100A8-deficient mice died during embryonic development, and S100A9-KO mice do not express S100A8 proteins [11]. Further, the stability of S100A8 protein and S100aA8/A9 function is effectively maintained by the presence of S100A9 [12]. Therefore, the present study aimed to explore the effect of S100A9 on sepsis-induced ALI.

The liver is the key organ that has an important role in the regulation of immune response and host defense. It consists of multiple cell types, including hepatocytes (HCs), sinusoidal endothelial cells (SECs), and Kupffer cells (KCs), which are involved in the processes of endotoxin and bacteria scavenging, detoxification, metabolism, immune functions, and substance synthesis [21]. Hepatic macrophages are a highly heterogeneous population that consists of self-renewing liver-resident Kupffer cells (KCs) located in the hepatic sinusoid and macrophages that are recruited from the bone marrow (BM) and the peritoneal cavity [22]. Recent studies have revealed that hepatic macrophages can secrete distinct sets of cytokines and chemokines, including TNF-α, IL-1β, and IL-6, which are the key mediators in systemic inflammatory response syndrome (SIRS) and the generation of hepatic acute-phase proteins during sepsis [22,23]. However, the pathogenesis of sepsis-induced acute liver dysfunction has not been completely elucidated. A global study revealed that S100A8/A9 plays critical roles in various diseases through different mechanisms. S100A8/A9 accelerates LPS-induced inflammatory responses and mortality through the activation of TLR4/RAGE and the production of TNF-α [1,2]. Moreover, genetic deletion or pharmacological inhibition of S100A8/A9 can inhibit the TLR4/NLRP3/IL-1β pathway, suppressing ischemia-induced granulopoiesis and improving cardiac dysfunction [16]. Deficiency of S100A8/A9 protects tubular epithelial cells from the UUO-induced apoptosis of tubular epithelial cells and renal fibrosis by inhibiting epithelial–mesenchymal transition [24]. S100A8 and S100A9 can function separately by binding to different receptors. S100A9 markedly enhances I/R-induced cardiomyocyte apoptosis and cardiac dysfunction through the TLR4/ERK/PPAR-mediated inhibition of mitochondrial function [15]. Cardiac-specific overexpression of S100A8 and S100A9 leads to a decrease in calcium flux and cardiac function in mice via the activation RAGE [18]. In contrast, inhalation of S100A8/A9 and S100A9 in mice decreases LPS-induced acute lung injury by enhancing the CXCL10-mediated infiltration of neutrophils and lymphocytes [25]. S100A8/A9 also increases the paracrine therapeutic effects of human mesenchymal stem cells by improving MI-induced inflammation, fibrosis, and cardiac dysfunction [26]. However, whether S100A9 is involved in LPS-induced hepatic injury and the underlying mechanism remains unclear. Here, using S100A9-KO mice and an S100A9 inhibitor in a CLP-induced sepsis model, we demonstrated for the first time that the ablation or inhibition of S100A9 significantly reduced CLP-induced mortality, liver dysfunction, cell apoptosis, inflammation, and oxidative stress in mice (Figure 2 and Figure 6), indicating that blocking S100A9 improves septic liver injury.

Sepsis-induced ALI is associated with significant alterations of mitochondrial function and energy metabolism. AMPK is a heterotrimer that consists of a catalytic α subunit and two regulatory β and γ subunits, which are allosterically activated by low ATP levels and high AMP levels [27,28]. This kinase is a key enzyme that activates mitochondrial biogenesis to improve cellular energy utilization by enhancing fatty acid (FA) oxidation and glucose uptake. AMPK also leads to autophagy activation, maintaining proper mitochondrial turnover [27,28]. Interestingly, AMPK is highly expressed in hepatocytes and other cell types and is required for the cellular energy utilization of normal mitochondrial function, the reduction of oxidative stress, and cell survival in the liver [29,30,31,32]. Notably, activation of AMPK by AICAR in male mice exerts beneficial effects on sepsis-induced hepatic injury, and these protective effects are related with the improvement in mitochondrial function [33]. AMPKα1 is highly expressed in hepatocytes. Male mice with hepatocyte-specific knockout of AMPKα1 (AMPKα1 KO) showed a higher mortality rate, higher blood IL-6 and TNF-α levels, and more severe liver and lung injuries. This was shown by the mitochondrial damage and decreased ATP levels compared to male WT mice 18 h after CLP, demonstrating that AMPKα1 is an important hepatoprotective factor during sepsis [34]. However, it is unknown whether AMPK activation is involved in the protection of S100A9 KO in CLP-induced ALI. To test this hypothesis, we assessed the protein levels of AMPK, ACC (a key modulator of FA oxidation) and GLUT4 (a critical regulator of glucose uptake) in the liver after sepsis. Our data indicated that the protein levels of p-AMPK, p-ACC and GLUT4 were markedly higher in the livers of S100A9-KO mice than in those of WT mice after CLP (Figure 3). Accordingly, both the mitochondrial OXPHOS capacity and ETS activity of complexes I and II as well as ATP production were dramatically increased in the livers of S100A9-KO mice compared with those of WT mice after CLP (Figure 3). In contrast, pharmacological inhibition of AMPK with Compound C dramatically abolished the S100A9 KO-mediated amelioration of liver dysfunction and injury and mitochondrial dysfunction after sepsis in vivo (Figure 5), suggesting that AMPK-mediated mitochondrial energy metabolism is essential for the attenuation of sepsis-induced liver injury. Overall, these results indicate that S100A9 contributes to CLP-induced liver injury, possibly by suppressing AMPK-dependent energy metabolism.

Accumulating data reveal that sepsis-induced liver dysfunction is related to poor clinical outcomes. However, no good therapeutic options are currently available to attenuate the most severe complications [1,2]. Early antibiotic therapy and supportive care are the main approaches for treatment at present. The burst of proinflammatory cytokines is a hallmark of sepsis, and it leads to subsequent multiple organ dysfunction, such as liver dysfunction, and death [1,2]. Thus, new anti-inflammatory therapies are needed. A recent study reported that apoptotic cell infusion can significantly suppress the production of multiple cytokines and chemokines and improve mitochondrial and glycolytic dysfunction, thereby resulting in an increase in mouse survival and the reversal of multiple organ dysfunction [35]. Interestingly, the increased level of S100A8/A9 in the blood of patients was highly associated with lower left ventricular ejection fraction (LVEF) [36]. Therefore, targeting S100A9 may be a novel therapeutic approach for preventing this disease. Indeed, blocking S100A8/A9 activity with small-molecule inhibitors (ABR-238901) or blocking antibodies significantly improves pathological conditions in various mouse models. For example, blocking S100A9 with the inhibitor ABR-238901 greatly attenuates the CLP-induced infiltration of neutrophils, formation of edema, and expression of Mac-1, CXCL1, CXCL2 and IL-6 in the plasma or lung and improves sepsis-induced lung injury [19]. The administration of Paq to mice markedly increases the survival rate and prevents pneumonia, effects accompanied by a reduction in viral loads, after SARS-CoV-2 infection in mice [20]. In addition, treatment with an S100A9 neutralizing antibody in mice highly attenuates myocardial I/R injury and ameliorates I/R-induced cardiac dysfunction [15]. Similarly, ABR-238901 treatment for 3 days significantly improves left ventricular function and cardiac output in mice 21 days after myocardial infarction [36,37]. Here, our study further demonstrated that the pharmacological inhibition of S100A9 with Paq effectively improved sepsis-induced liver injury and mitochondrial dysfunction in CLP-treated mice (Figure 6 and Figure 7), suggesting that blocking S100A9 may be a new therapeutic approach for sepsis-induced liver injury.

In conclusion, this study reveals the key role of S100A9 in promoting sepsis-induced hepatic dysfunction and injury, partially by activating AKT and inhibiting AMPK-mediated mitochondrial energy metabolism. Our data also highlight that S100A8/A9 may be a new therapeutic target for sepsis-induced liver injury. Future studies will have to specifically examine the role of the myeloid-specific deletion of S100A8/A9, particularly S100A8 in the pathogenesis of sepsis-induced hepatic dysfunction, define the precise mechanism by which S100A8/A9 inhibits the activation of AMPK signaling in the liver, and determine whether targeting S100A9 presents a new opportunity for the treatment of sepsis and multiple organ failure. Further study is required to investigate how S100A9 is upregulated in CLP-treated livers.

## 4. Material and Methods

### 4.1. Animals and Treatment

WT C57BL/6J mice were purchased from The Jackson Laboratory (Sacramento, CA, USA). Global S100A9 knockout (KO) mice (KOCMP-20202-S100A9-B6N-VA) were generated and fully backcrossed onto the C57BL/6 background more than 10 generations (Cyagen Biosciences Inc., Santa Clara, CA, USA) as described previously [15]. S100A9-KO mice were viable. Genotyping primers for S100A9 KO were as follows: forward, 5′-GTATATGTGGAGGGAAGCTGTCTC-3′; and reverse, 5′-GTGAAAGGAGGCAGAAAGGACATG-3′. Eight- to ten-week-old male WT and S100A9-KO mice were maintained under a 12:12 h light–dark photocycle throughout the period of study.

### 4.2. Cecal Ligation and Puncture Operation

Male WT (*n* = 200) and S100A9-KO (*n* = 90) mice (8–10 weeks old, 22–26 g) were adaptively fed for 7 days, and a polymicrobial sepsis model was established by cecal ligation and puncture (CLP) as described [38]. In the CLP group, the mice were anesthetized with 2.5% tribromoethanol (0.01 mL/g; Sigma, St. Louis, MO, USA), a 1 cm midline laparotomy was performed, and the cecum was fully exposed, ligated and punctured through both surfaces once with a 21-gauge needle. Then, the cecum was returned to the peritoneal cavity, and the incision was closed with a 4-0 nylon suture. The control mice did not undergo ligation and puncture. To confirm the impact of S100A9 inhibition on CLP-induced liver injury, WT mice were injected intraperitoneally (IP) with Paquinimod (HY-100442, MCE, Princeton, NJ, USA) at 5–20 mg/kg or control vehicle (castor oil) before and after CLP operation. The AMPK inhibitor Compound C (HY-13418, MCE, Princeton, NJ, USA) was dissolved in normal saline at 20 mg/kg and IP-injected 12 h and 1 h prior to CLP surgery. The mice were then sacrificed 24–72 h after CLP. Liver and blood were harvested to examine the function, histology, and mRNA and protein levels.

### 4.3. Biochemical Measurements

Serum levels of alanine aminotransferase (ALT; C009-2-1) and aspartate aminotransferase (AST; C010-2-1) were analyzed as indices of liver function; serum and tissue levels of triglyceride (TG; A110-1-1) and free fatty acids (FFAs; A042-2-1) were measured to evaluate lipolysis. All colorimetric assay kits were obtained from Nanjing jiancheng (Nanjing, China), and the experiments were performed based on the manufacturer’s protocols.

### 4.4. Histological Examinations

Hepatic specimens were fixed in 4% paraformaldehyde and embedded in paraffin. Liver sections (4-µm thick) were cut serially and stained with an H&E staining kit based on the instructions. Immunohistochemistry was performed on the sections with anti-S100A9 (73425, CST, Danvers, MA, USA) or anti-CD68 (ab125212, Abcam, Cambridge, MA, USA) antibodies. Immunofluorescent staining was conducted with anti-S100A9 (73425, CST) and anti-F4/80 (6640, CST) antibodies to assess the localization of S100A9 in macrophages. The histological score of H&E tissue staining was determined based on four grades according to a previous study [39].

To evaluate liver cell apoptosis, tissue cryosections at 6 µm thickness were stained by using a TUNEL Apoptosis Detection kit (Roche, Basle, Switzerland) based on previously described protocols [40]. The ratio of TUNEL-positive cells to DAPI-positive nuclei was calculated and presented as the percentage of apoptotic cells for each group. For superoxide analysis, cryosections were stained with 1 µmol/L dihydroethidine (DHE, Sigma, St. Louis, MO, USA) in a PBS buffer (pH 7.4) for 30 min at 37 °C, as described previously [41]. Fluorescence images were obtained from 6–8 random fields for each sample with a Nikon microscope (Nikon, Tokyo, Japan).

### 4.5. Quantitative Real-Time PCR Detection of mRNA Expression in the Liver

The liver tissues were ground in a tissue-crushing apparatus (OMNI International, New York, NY, USA). Total RNA from livers was purified using a TRlZOL reagent kit (9109, TAKARA, Tokyo, Japan), and total 1 μg RNA was-reverse transcribed to cDNA with a PrimeScript RT reagent kit (RR047A, TAKARA). Then, qPCR was performed on a QuantStudio^®^ 3 system using primers specific for interleukin (IL)-1β, IL-6, TNF-α, NOX1, and NOX2. GAPDH was used as the internal control. Finally, the relative mRNA expression levels of genes were statistically analyzed with 2^−ΔΔCt^. All the primers for the target genes were synthesized by Sangon Biotech Co., Ltd. (Shanghai, China) and prepared to use the following primers: S100A8 (5′-GGA GTT CCT TGC GAT GGT GA-3′ and 5′-GGC CAG AAG CTC TGC TAC TC-3′), S100A9 (5′-ATA CTC TAG GAA GGA AGG ACA CC-3′ and 5′-TCC ATG ATG TCA TTT ATG AGG GC-3′), IL-1β (5′-CTT CCC CAG GGC ATG TTA AG-3′ and 5′-ACC CTG AGC GAC CTG TCT TG-3′), IL-6 (5′-GCT ACC AAA CTG GAT ATA ATC AGG A-3′ and 5′-CCA GGT AGC TAT GGT ACT CCA GAA-3′), TNF-α (5′-ATG GCC TCC CTC TCA TCA GT-3′ and 5′-CTT GGT GGT TTG CTA CGA CG-3′), NOX1 (5′-CCC ATC CAG TCT CCA AAC ATG AC-3′ and 5′-ACC AAA GCT ACA GTG GCA ATC AC-3′), NOX2 (5′-GGG AAC TGG GCT GTG AAT GA-3′ and 5′-CAA TTG TGT GGA TGG CGG TG-3′), GAPDH (5′-CAT CAC TGC CAC CCA GAA GAC TG-3′ and 5′-ATG CCA GTG AGC TTC CCG TTC AG-3′).

### 4.6. Immunoblotting Measurement of Protein Levels

The liver tissues were removed and fully lysed using a RIPA lysis buffer. The homogenates were centrifuged at 13,300 rpm at 4 °C for 15 min. The protein concentrations in the supernatant were quantified by the BCA method (Thermo Fisher, Carlsbad, CA, USA). A total of 50 μg of protein per sample was resolved by 7.5–15% SDS–PAGE and transferred onto a PVDF membrane. Next, the membranes were blocked in 5% skim milk for 1 h at room temperature and incubated with the appropriate primary antibodies at 4 °C overnight, and then incubated with the secondary antibodies at room temperature for 1 h. Finally, the membranes were developed on a FluorChem M System (PerkinElmer, Waltham, MA, USA). The intensities of protein bands were quantified with NIH Image J version 1.48, and the relative levels of the corresponding proteins were calculated in each group. The primary antibody against S100A9 (ab242945, 1:1000) was from Abcam (Cambridge, MA, USA). Antibodies against S100A8 (47310; 1:1000), p-AMPK (2535; 1:1000), AMPK (5831; 1:1000), p-AKT (4060; 1:2000), AKT (4691; 1:1000), ACC (3662; 1:1000) and β-actin (4970; 1:1000) were from Cell Signaling Technology (CST, Danvers, MA, USA). Antibodies against GLUT4 (66846-1-Ig; 1:3000), Bax (50399-2-Ig; 1:7000) and Bcl-2 (12789-1-AP; 1:2000) were obtained from Proteintech Group (Chicago, IL, USA).

### 4.7. Detection of Mitochondrial Respiratory Function

Mitochondrial respiratory function was detected in liver tissues with an Oroboros Oxygraph-2k (Innsbruck, Austria) in a thermostat-controlled chamber as described [42]. Briefly, fresh liver tissues (10 mg) were cut into small pieces and homogenized with a Dounce tissue grinder in 400 mL mitochondrial respiration solution (MiR05), and the process was performed on ice. Then, we used 70 μL liver tissue homogenates to measure mitochondrial respiratory function. After a 2 min equilibration, we first titrated 2 μM safranin O to measure the mitochondrial membrane potential (MMP), and mitochondrial respiration was examined by following SUIT. Briefly, 20 mM glutamate (G), 4 mM malate (M) and 1.5 mM ADP d, were added in sequence to determine the state 4 (leak state) and state 3 (phosphorylation) respiration of complex I. Succinate (S, 10 mM) was added to determine the state 3 respiration of complex II. Between CI and CII, damage to the outer mitochondrial membrane was defined as the percentage increase after the addition of cytochrome c (C, 10 μM), and samples with values over 20% were removed. Then, 0.03 μM oligomycin (Omy) was titrated to measure ATP production. FCCP (U, final concentration of 2–3 μM) was then administered to determine the maximal uncoupled respiratory capacity of the electron transfer system (ETS), and rotenone (Rot, 1 μM) was administered for CII ETS. Finally, antimycin A (AA) was injected to detect residual oxygen consumption (ROX). ATP production was measured as 1-(OCR after the addition of G, M, D, C, S, and Omy)/(OCR after the addition of G, M, D, C, and S), and MMP was inversely related to the fluorescence of safranin O, being quenched in the mitochondrial matrix in hyperpolarized mitochondria [42,43]. The MiR05 consists of 20 mM HEPES, 0.5 mM EGTA, 3 mM MgCl_2_·6H2O, 110 mM D-sucrose, 60 mM lactobionic acid (LBA), 20 mM taurine, 10 mM KH_2_PO_4_, and 1 g/L BSA (fatty acid free). All data were recorded with DatLab acquisition software 5.2 (Oroboros Instruments, Innsbruck, Austria).

### 4.8. Transmission Electron Microscopy (TEM)

Liver tissues were cut into 1 mm^3^ pieces, and were fixed in 2.5% glutaraldehyde for 2 h at 4 °C. The pieces were then post-fixed in 1% osmium tetroxide in phosphate buffer (PB) for no more than 2 h, and washed 3 times with PB. After washing, the samples were dehydrated, immersed and finally embedded overnight at room temperature. The tissues pieces were cut with an ultramicrotome. The sections (50–70 nm) were stained with uranyl acetate and lead citrate, and were then photographed with an HT7700 transmission electron microscope (HITACHI, Tokyo, Japan) at 100 kV. The total number of mitochondria and the number of abnormal or impaired mitochondria were analyzed in 5–6 cells in 3 sections from each sample with the NIH Image J software version 1.48.

### 4.9. Statistical Analysis

All the results are presented as the mean ± standard error of the mean (SEM) and were statistically analyzed using SPSS version 25.0 (IBM, Armonk, YK, USA). A normality test Shapiro–Wilk was used to test if the results were normally distributed. If the results followed a normal distribution, Student’s *t* test was used to analyze the significance of the differences between 2 groups, and if not, the Mann–Whitney test was used. One-way ANOVA was used to analyze differences among 3 or more groups. However, when the results were not normal or equal variances were not assumed (Welch test), Kruskal–Wallis H was used. The Kaplan–Meier method and log-rank tests were conducted to analyze the survival rates of the mice after the sham or CLP operation. *p* < 0.05 was considered significant.

## Data Availability

The datasets generated during and/or analysed during the current study are available from the corresponding author on reasonable request.

## Abbreviations

CLP	Cecal ligation and puncture:
TNF-α	Tumor necrosis factor alpha
IL-1β	Interleukin-1β
IL-6	Interleukin-6
ATP	Adenosine triphosphate
AMPK	Adenosine 5′-monophosphate (AMP)-activated protein kinase
AKT	Protein kinase B
ACC	Acetyl-CoA carboxylase
GLUT4	Glucose transporter 4
ETS	Electron transfer system
OXPHOS	Oxidative phosphorylation
