# Correction: Zhang et al. Deficiency of S100A9 Alleviates Sepsis-Induced Acute Liver Injury through Regulating AKT-AMPK-Dependent Mitochondrial Energy Metabolism. *Int. J. Mol. Sci*. 2023, *24*, 2112

**DOI:** 10.3390/ijms26030935

**Published:** 2025-01-23

**Authors:** Yanting Zhang, Feng Wu, Fei Teng, Shubin Guo, Huihua Li

**Affiliations:** Beijing Key Laboratory of Cardiopulmonary Cerebral Resuscitation, Department of Emergency Medicine, Beijing Chaoyang Hospital, Capital Medical University, Beijing 100020, China; yanting953@mail.ccmu.edu.cn (Y.Z.);

In the original publication [1], there was a mistake in Figure 4D as published. The image of the H&E staining in the CLP-KO+CC group in Figure 4D was shown incorrectly during the manuscript preparation. The correct version of Figure 4D is included below. The authors state that the scientific conclusions are unaffected. This correction was approved by the Academic Editor. The original publication has also been updated.

## Figures and Tables

**Figure 4:**
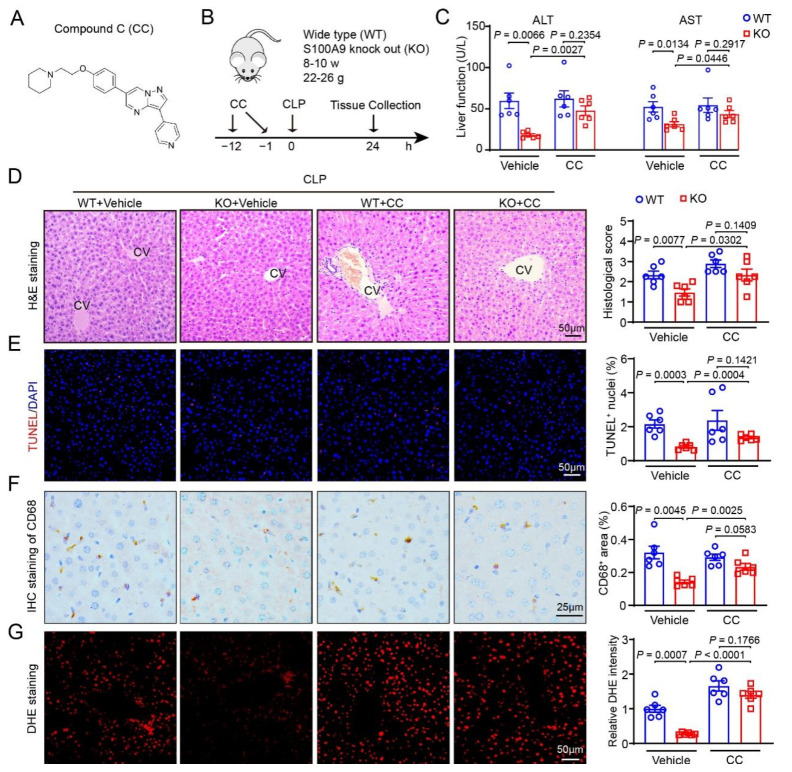
Inhibition of AMPK reverses S100A9-KO-mediated protection of CLP-induced liver dysfunction. (**A**) Diagram of AMPK inhibitor Compound C (CC). (**B**) Schematic diagram of WT or S100A9-knockout (KO) mice treated with CC and CLP operation for 24 h. (**C**) The levels of serum ALT and AST in each group (*n* = 6). (**D**) H&E staining of liver sections (left), and quantification of the histological score (right, *n* = 6). (**E**) TUNEL (red) and DAPI (blue) staining of liver sections (left), and quantification of TUNEL^+^ nuclei (right, *n* = 6). (**F**) Immunohistochemical staining of liver sections with anti-CD68 antibody (left), and quantification of the CD68^+^ area (right, *n* = 6). (**G**) DHE staining of liver sections to measure superoxide production (left), and quantification of fluorescence intensity (right, *n* = 6). Data are shown as mean ± SEM, and *n* represents the number of mice in each group.
